# Evaluation of Bioinformatics Approaches for Next-Generation Sequencing Analysis of microRNAs with a Toxicogenomics Study Design

**DOI:** 10.3389/fgene.2018.00022

**Published:** 2018-02-06

**Authors:** Halil Bisgin, Binsheng Gong, Yuping Wang, Weida Tong

**Affiliations:** ^1^Department of Computer Science, Engineering, and Physics, University of Michigan–Flint, Flint, MI, United States; ^2^Division of Bioinformatics and Biostatistics, National Center for Toxicological Research (FDA), Jefferson, AR, United States

**Keywords:** miRNA, next generation sequencing (NGS), miRNA-seq, miRNA profiling, toxicogenomics, thioacetamide

## Abstract

MicroRNAs (miRNAs) are key post-transcriptional regulators that affect protein translation by targeting mRNAs. Their role in disease etiology and toxicity are well recognized. Given the rapid advancement of next-generation sequencing techniques, miRNA profiling has been increasingly conducted with RNA-seq, namely miRNA-seq. Analysis of miRNA-seq data requires several steps: (1) mapping the reads to miRBase, (2) considering mismatches during the hairpin alignment (windowing), and (3) counting the reads (quantification). The choice made in each step with respect to the parameter settings could affect miRNA quantification, differentially expressed miRNAs (DEMs) detection and novel miRNA identification. Furthermore, these parameters do not act in isolation and their joint effects impact miRNA-seq results and interpretation. In toxicogenomics, the variation associated with parameter setting should not overpower the treatment effect (such as the dose/time-dependent effect). In this study, four commonly used miRNA-seq analysis tools (i.e., miRDeep2, miRExpress, miRNAkey, sRNAbench) were comparatively evaluated with a standard toxicogenomics study design. We tested 30 different parameter settings on miRNA-seq data generated from thioacetamide-treated rat liver samples for three dose levels across four time points, followed by four normalization options. Because both miRExpress and miRNAkey yielded larger variation than that of the treatment effects across multiple parameter settings, our analyses mainly focused on the side-by-side comparison between miRDeep2 and sRNAbench. While the number of miRNAs detected by miRDeep2 was almost the subset of those detected by sRNAbench, the number of DEMs identified by both tools was comparable under the same parameter settings and normalization method. Change in the number of nucleotides out of the mature sequence in the hairpin alignment (window option) yielded the largest variation for miRNA quantification and DEMs detection. However, such a variation is relatively small compared to the treatment effect when the study focused on DEMs that are more critical to interpret the toxicological effect. While the normalization methods introduced a large variation in DEMs, toxic behavior of thioacetamide showed consistency in the trend of time-dose responses. Overall, miRDeep2 was found to be preferable over other choices when the window option allowed up to three nucleotides from both ends.

## Introduction

MicroRNAs (miRNAs), which are ∼22 nucleotides (nts) short non-coding RNAs, comprise a class of gene regulatory elements that have a major effect on the stability and translation of messenger RNAs (mRNAs). The regulatory effect of miRNAs has been observed in the development stages, tissue-specific expressions, disease states and toxicological effects. Recent studies reveal that the role of miRNAs is more profound than it was suspected (Zhang and Su, 2009) as they target ∼30% of all genes in animals (Lewis et al., 2005) and contribute to a spectrum of human diseases with potential therapeutic options (Chen and Verfaillie, 2014; Finch et al., 2014). For example, in cancer research, miRNAs (e.g., miR-374a) can serve as prognostic markers (Vosa et al., 2011). Despite their low abundance in body fluids, miRNAs have been studied as non-invasive biomarkers for diseases (Cortez et al., 2011).

Due to their crucial role in regulatory processes, miRNAs are also associated with toxicity (An and Hwang, 2014). As toxicogenomics studies aim to elucidate the relation between toxicant exposure and genome-wide gene expression patterns, miRNAs play an important role as key mediators in toxicological research (Lema and Cunningham, 2010). miRNA profiling associated with environmental toxicants, industrial chemicals, and drugs have been studied on different organisms such as humans, mice, and rats (Marsit et al., 2006; Fukushima et al., 2007; Pogribny et al., 2007; Sathyan et al., 2007; Shah et al., 2007). While most of these studies apply qPCR or microarray techniques, RNA sequencing (RNA-seq) has gained a larger role in miRNA profiling due to its use of next-generation sequencing, namely miRNA-seq.

RNA-seq has several advantages over conventional profiling techniques such as qPCR and microarrays, particularly as its cost has lowered in recent years. For example, RNA-seq is not bound with the pre-defined genes to be assayed, which increases the number of genes to be detected and reveals new transcripts (Mestdagh et al., 2014). In addition, RNA-seq has been demonstrated with low variation between platforms compared to the conventional techniques (Consortium, 2014). Furthermore, RNA-seq has a better dynamic detection range (Zhao et al., 2014). However, the choice of analysis methods has been a crucial step for RNA-seq data analysis and subsequent biological interpretation. In-depth analyses of RNA-seq pipelines for mRNA expression analysis were reported but a comprehensive analysis of various bioinformatics pipelines for miRNA-seq applied in toxicogenomics study has yet been conducted. A toxicogenomics study design usually involves both dose- and time-dependent features. It is important that the choice of miRNA-seq analysis methods should not shadow the dose and time-dependent treatment effect. Therefore, this study aims to assess various miRNA pipelines for miRNA profiling exposed to a toxicant with multiple dose and time points. Of note, this study is to assess the joint effect of various parameters involving mapping, quantification, and selection of the profiling tool, rather than novel discoveries and isoform identification.

There are several tools for miRNA-seq data analysis and some have been evaluated for different purposes in previous studies. Li et al. (2012) compared eight tools: miRDeep (Friedlander et al., 2008), miRanalyzer (Hackenberg et al., 2009), miRExpress (Wang et al., 2009), miRTRAP (Hendrix et al., 2010), DSAP (Huang et al., 2010), mirTools (Zhu et al., 2010), MIReNA (Mathelier and Carbone, 2010), miRNAkey (Ronen et al., 2010), and mireap (Sourceforge, 2015). They selected three data sets (*Caenorhabditis elegans*, *Gallus gallus*, and human embryonic stem cells) to evaluate the sensitivity, accuracy and potential for novel miRNA discovery of these tools. They provided a preference list of tools for these three organisms in predicting novel miRNAs. To observe miRNA response to acute nerve crush, Metpally et al. (2013) conducted an analysis on miRDeep2 (Friedländer et al., 2012), miRExpress, and miRNAkey which was followed by the computation of DEMs by DESeq (Anders and Huber, 2010) and EdgeR (Robinson et al., 2010). miRDeep2 was consistently reported to identify the highest number of DEMs. Whereas the former study was focused on novel miRNA discovery in comparison, the latter measured the DEM concordance of tools and provided novel miRNA predictions for a neurological disease. However, to the best of our knowledge, such an extensive evaluation on bioinformatics choices for toxicogenomics data has not been conducted. It is also important to note that miRDeep2 is the evolved version of miRDeep while sRNAbench has recently become the latest version of miRanalyzer. Their parameters in the earlier versions were not extensively studied to confirm whether such changes cause any major downstream perturbations. This not only necessitates revisiting the current versions of these tools and comparing with other tools, but also provides the opportunity for a new study design with a toxicogenomics focus.

We propose a toxicogenomics study design that is based upon time-dose resolution of a toxic treatment to addresses questions. The study conducted focused on the parameter choices of selected tools for miRNA profiling as it may have an impact on DEMs that plays a central role in toxicogenomics for mechanistic interpretation of toxicity. Whereas an ideal design would require a comparison based on a ground truth set of DEMs, we introduced a method that used the number of DEMs as a means in the absence of such ground truth. In specific, we examined the variability in the number of quantified miRNAs and detected DEMs under certain conditions for selected profiling tools: miRDeep2, sRNAbench, miRExpress, and miRNAkey. For a comprehensive analysis including time-dose response, we employed miRNA-seq data acquired through rat liver samples that were treated by thioacetamide, a well-known carcinogen (Fitzhugh and Nelson, 1948), in four time points and at three dose–levels with their concurrent control. The dataset allowed us to measure the effect of treatment as we changed the parameters of the tools as well as the normalization methods. Especially important for downstream analysis was the use of change in the number of DEMs as a measure to assess the stability of a tool as well as the competition between treatment and parameter combinations.

## Materials and Methods

### Data Acquisition and Processing

The data used in this analysis is reported from our lab (Dweep et al., 2017) and available in the GEO repository (GSE87446)^1^. Briefly, thioacetamide was administered to rats for 3, 7, 14, and 28 days with 4.5, 15, and 45 g/kg, daily. On a concurrent basis, another group of healthy rats were kept as a control group. Being the main target organ in this project, liver samples obtained from both the treated and the control groups were further processed for RNA isolation and the isolates exceeding the RNA integrity number (9.0) were sent out for sequencing.

Following the separation of miRNAs from other RNAs, sequencing was conducted on the Illumina HiSeq 2500 platform and sequencing data were de-multiplexed. Resulting fastq files were inspected for quality control purposes. Lanes having low sequence depth were discarded, which in turn caused the loss of one control sample for 3 days. Other discarded lanes only led to the excise of some technical replicates, but did not cause any further exclusion of time-dose points and control samples. Average Phred scores for the sustained lanes varied from 38 to 40. Before employing the profiling tools on the reads, we trimmed the Illumina 3′ adapter by using *fastx_clipper* in the FASTX-Toolkit (Metpally et al., 2013).

### miRNA Profiling Tools

Several miRNA-seq tools have been reported with different underlying algorithms and running environments, i.e., web server vs. a stand-alone application. These tools usually provide both expression profiling and miRNA prediction. In this study, we selected four popular tools that can run on a local system since uploading big data sets to the web-based tools is time-consuming and brings further limitations. We used the 21st release of miRBase (Kozomara and Griffiths-Jones, 2014) as the reference database for all tools in the alignment step. In **Table 1**, we summarize the features of the tools by also giving the applicability information of an option and the number of choices we examined for parameters. Most variations are for windowing (more than four options for each tool) and quantification (two options for each tool).

**Table 1:** Summary of tool features along with their options.

	Alignment algorithm	Genome mapping	Windowing	Quantification	Total parameter
miRDeep2	Bowtie	Y	Y(6)	Y(2)	12
sRNAbench	Bowtie	Y	Y(6)	Y(2)	12
miRExpress	SW	NA	Y(4)	NA	4
miRNAkey		NA	NA	Y(2)	2

*SW, Smith–Waterman algorithm; Y, yes; NA, not applicable.*

During the biogenesis of miRNAs, there might be errors in the Dicer process and therefore some mismatches can be tolerated out of the mature sequence during mapping. While sRNAbench introduces the terms *WinUp* and *WinDown*, miRDeep2 allows such tolerance by –e and –f parameters in the *quantifier.pl* module. These parameters refer to a window size in both directions of mature sequence and we used windowing as a profiling parameter that took values in {0, 3, 5} and {0, 3} for 3′- and 5′-UTR directions, respectively. Hence, windowing introduces six combinations for the profiling step. However, this was not applicable to miRNAkey and miRExpress. While miRNAkey did not support any extensions, miRExpress worked with the total window size regardless of the direction. For instance, if the window size (or tolerance parameter –g) is set to 8, miRExpress might be allowing 2 nts from 5′ UTR and 6 nts from 3′ UTR. Alternatively, it might be tolerating 8 nts from 5′ UTR, but none toward 3′ UTR. Finally, those six combinations were only tested for miRDeep2 and sRNAbench as a part of their evaluations.

Read fragments that can be mapped to multiple miRNAs are handled differently by all four tools. In this step, tools distribute mapped reads to known miRNAs in their own way and output the expression values accordingly. miRNAkey employs SEQ-EM for additional preferences for the allocation of multiply aligned reads. In this step, we had both the multiple mapping and single mapping values for sRNAbench and miRDeep2 which were computed on the basis of their outputs.

#### miRDeep2

miRDeep2 (Friedländer et al., 2012) is a software package which consists of Perl scripts and is the updated version of miRDeep. The use of its components allows both expression profiling and identification of novel miRNAs. Preprocessing of the reads is accomplished through its *mapper.pl* script while quantification is achieved by *quantifier.pl*. For the novel miRNA identification, *mirDeep2.pl* is utilized. miRDeep2 offers various options for each script. Since the underlying alignment tool is Bowtie, alignment parameters can either be set on the command line or modified in the source code. Default parameters consider mismatches outside the mature sequence for both 3′ and 5′ directions through “windowing,” that is, tolerating mismatches from both ends. miRDeep2 also introduces further flexibility when counting the mapped reads in quantification, i.e., single mapping, multiple mapping, and unique counts.

#### sRNAbench

Similar to miRDeep2, sRNAbench is also an improved version of an earlier tool, i.e., miRanalyzer, in which novel features, such as genome and library mapping, have been added. In addition, its prediction capability has been improved for novel miRNAs and isomiR support. It consists of different modules such as preprocessing, mapping, and profiling/detection. For the mapping module, it also employs Bowtie. With a variety of output options, it can also provide a differential expression analysis by using EdgeR. With the parameters *WinUp* and *WinDown* it provides the windowing option that sets a tolerance value for the mismatches toward 3′- and 5′-UTR. Moreover, the user has the option to incorporate genome mapping in the pipeline.

#### miRExpress

Unlike miRDeep2 and sRNAbench, miRExpress (Wang et al., 2009) employs the Smith-Waterman (SW) algorithm (Smith and Waterman, 1981) for the alignment. It comes with different constituents including preprocessing steps such as adapter removal. miRExpress also differs in that it does not need genome mapping, directly mapping the reads to the known miRNAs documented in miRbase instead. While the tools mentioned above allow for a tolerance value in both directions of the mature sequence, miRExpress only allows a total number of mismatches from either direction. That is, the user sets a window size through the windowing option and miRExpress tolerates any number of mismatches that do not exceed this size.

#### miRNAKey

Another profiling tool which directly maps the reads to known miRNAs is miRNAKey (Ronen et al., 2010). It comes with a stand-alone Java package that can be used either on the command line or through a GUI. It further allows for adapter removal and performs alignment by using BWA (Smith and Waterman, 1981). As for the counting option in **Table 1**, it utilizes the SEQ-EM algorithm (Pasaniuc et al., 2011) to optimize the distribution of multiply aligned reads.

### Applicability of Parameters across Tools

Although the tools mentioned above have unique features, they also have similar parameters that can be compared to some extent. While tool-specific parameters were used for internal evaluation of each tool by measuring the variability and treatment effect in the number of DEMs, compatible ones were utilized for a cross-tool comparison. Windowing and quantification with two counting options (single and multiple) served as two factors for a side-by-side comparison of miRDeep2 and sRNAbench.

### Side-by-Side Comparison of sRNAbench and mirDeep2

Since both tools detected around 500 miRNAs with expression values greater than zero, we ranked miRNAs in a decreasing order based on those quantifications for every parameter combination. Thus, we compared the agreement between these tools at each increment of 5 in the ordered lists under the same conditions. This procedure enabled us to follow the concordance in terms of percentages as we cover the whole list for a given parameter set.

We conducted a similar analysis for the DEMs obtained from different tools under the same parameter set and normalization method (*upper quartile*) in the EdgeR package. However, the number of DEMs was much lower than the detected miRNAs and varied drastically from one treatment to another. For these reasons, we performed this analysis for the treatment points for which we obtained more than 35 DEMs and considered miRNAs with a *p*-value less than 0.05 regardless of their fold changes.

### Pairwise Concordance of Tools

In this part of the study, we measured the agreement between tools by computing the Jaccard similarity. In other words, we normalized the overlapping quantity by the union of two miRNA sets that were obtained using different parameters. In the quantification stage, 24 parameters (2 tools × 6 windowing options × 2 quantification options) were used to achieve this pairwise evaluation. The resulting similarity values were used to generate hierarchical clusters that provide a visual aid to distinguish the impact of parameter selections.

### Analysis of Variance for Quantified and Differentially Expressed miRNAs

Two profiling elements (windowing and quantification) have become factors with different levels for the analysis of variance (ANOVA). Due to the available options for windowing, its effect was measured based on six levels that will be detailed later. Quantification, however, was limited to two levels. In this particular design, our dependent variables were the number of DEMs for which we measured the impact of each profiling element in terms of variance. In the following steps, we included the selection of the tool as another factor, which accounted for the effect of any tool on the variance. Then, we incorporated treatment elements as another set of factors. Specifically, we defined three more variables: (i) time, (ii) dose, and (iii) time-dose interaction, which introduced 4, 3, and 12 levels, respectively. In order to see the normalization effect in the number of DEMs, we further added normalization into the equation with three methods [TMM (Robinson and Oshlack, 2010), RLE (Bullard et al., 2010), and upper quartile (Bullard et al., 2010)] in EdgeR while also observing the changes without any normalization (NO).

### Time-Dose Response

All the parameters above are expected to have an effect on the downstream. Beside the magnitude of such an effect, we are also interested in whether the trend in time-dose response is affected by the choice of parameters. Therefore, we calculated the correlation in the number of DEMs across different treatments for every pair of parameter combinations.

## Results

### Study Design

**FIGURE 1:**
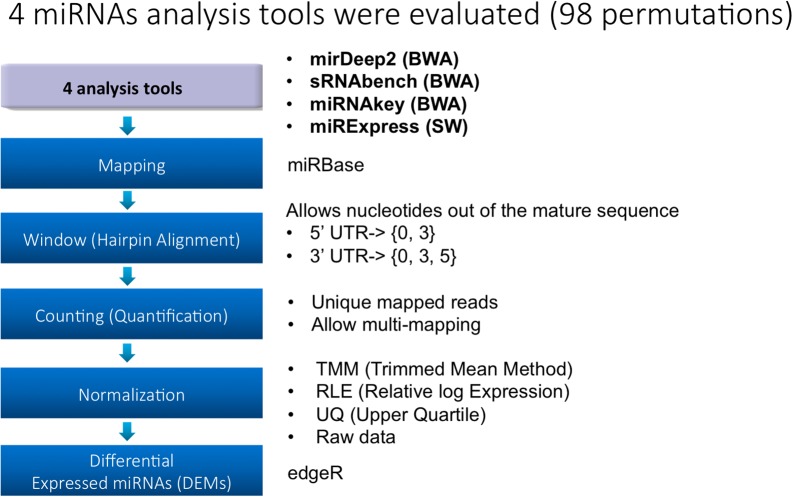
Study design.

Rat liver miRNA data from thioacetamide treatment at multiple doses and time points provided a two dimensional resolution of the treatment effect in both dose and time directions. As such, the number of DEMs across treatments has become a means for assessing the parameter effect and its association with the treatment pattern. As depicted in **Figure 1**, four analysis tools were selected. For each tool, parameter choices constituting the next three steps (i.e., mapping, hairpin alignment and counting) introduce further options. For each treatment point, we employ the same parameter combinations for miRNA quantification and DEM detection. Due to the incompatibility issues, 30 parameter combinations shown in **Table 1** are tested based on their applicability through the pipeline in **Figure 1**. Once the miRNAs are quantified, each tool is evaluated within its own parameter space by measuring its sensitivity in the number of DEMs and the impact on the treatment effect. As described below, these evaluations are used as selection criteria for further investigation of miRDeep2 and sRNAbench. Finally, we compare these two tools along with their parameter combinations in terms of miRNA quantification and DEM detection that introduces four normalization choices.

### Parameter Setting Introduced a Large Variation in miRExpress and miRNAKey

Selection of a tool is followed by the appropriate option to be employed with parameter manipulations for miRNA profiling as listed in **Figure 1**. For instance, the user needs to decide whether a tolerance value should be set for mismatches in 5′ UTR and 3′ UTR if applicable. Here, we introduce these parameters by discussing the applicability issues.

Each tool comes with own its features, underlying algorithm, and parameter set. Aiming for a comparable analysis of parameter combinations, we considered all parameters that can be manipulated and observed their responses (in terms of number of DEMs) to such changes. For a fair comparison, we performed the DEM calculations under the same normalization method (upper quartile) in EdgeR. Then, we computed the variance in the number of DEMs as we changed the parameters of each tool by which we obtained the coefficient of variation (CoV). It was observed that miRExpress showed higher CoV due to the change in its tolerance (windowing) parameter. Since it was not employing any other parameters, only the tolerance value for windowing was tuned and it was found to be more sensitive to the changes (**Supplementary Figure S1**).

**FIGURE 2:**
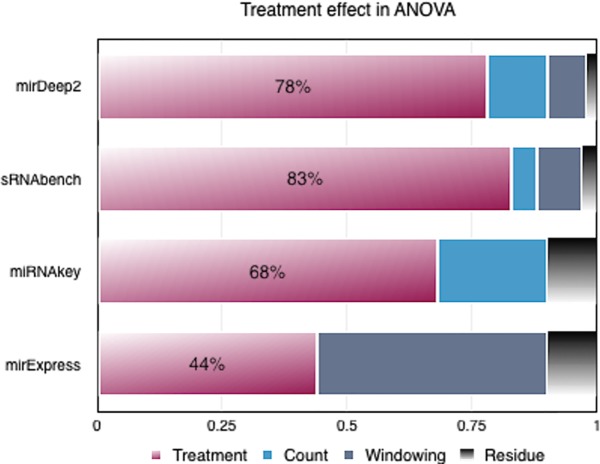
Variance analysis on the number of differentially expressed miRNAs (DEMs) produced by each tool.

miRNAkey had a comparable CoV with sRNAbench mainly due to its SEQ-EM option when counting the mapped reads. However, when we performed ANOVA analysis on the number of DEMs to compare the impact of tools with their parameters on the treatment effect, we observed that both miRExpress and miRNAkey shadowed the treatment effect more as illustrated in **Figure 2**.

On the other hand, in the case of miRDeep2 and sRNAbench, neither their individual parameters, nor their combined parameters dominated the treatment effect in terms of variance. Therefore, we chose miRDeep2 and sRNAbench for an in-depth investigation of parameter effect on the number of DEMs, which is an essential part of downstream analysis. Compatible options between these two tools also gave us the opportunity to perform further variance analyses.

### miRNA Quantification by sRNAbench and miRDeep2

#### Concordance for sRNAbench and miRDeep2

**FIGURE 3:**
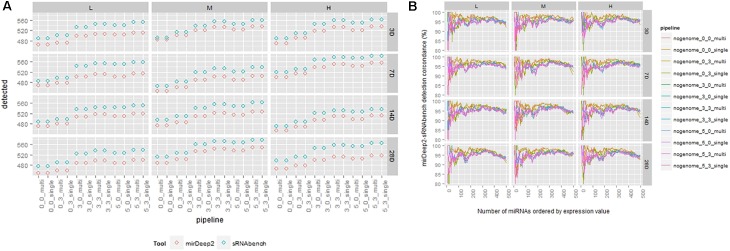
**(A)** Number of detected miRNAs for each tool under the same pipeline parameters. **(B)** Detection comparison in sorted lists from sRNAbench and miRDeep2 under the same parameter sets.

In this study, we considered a miRNA that has a non-zero expression value as detected. Then, we compared the total number of detected miRNAs for both tools and computed the overlaps in the ranked lists based on expression values. For varying profiling parameters, we counted the number of miRNAs that have an expression value greater than zero (**Supplementary Table S1**). This analysis showed that sRNAbench mostly reported more miRNAs than miRDeep2 (**Figure 3A**). Nevertheless, they had a high overlap of around 95% for each parameter combination. For each treatment point, we compared their sorted lists which were in decreasing order w.r.t. expression values. Excepting their top positions, which still showed an agreement around 80%, both tools were able to capture the same miRNAs within the top 500 candidates (**Figure 3B**).

#### Variance Analysis

In the analyses above, we presented the agreement across different bioinformatics choices pictorially. However, a holistic view is still needed to quantify the level of such impact and to further assess the significance of a choice against treatment effect or toxic exposure. Therefore, we performed ANOVA for various scenarios.

**FIGURE 4:**
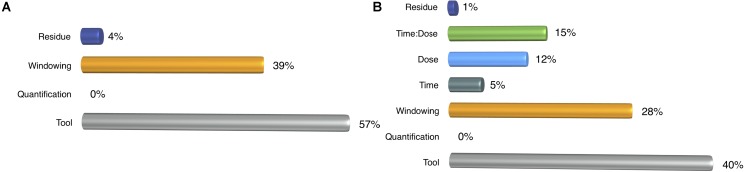
ANOVA results for detection rate. **(A)** Profiling parameters **(B)** treatment factors are added to the analysis.

For the detection task, we performed ANOVA for two cases based on: (i) profiling parameters, (ii) profiling parameters with treatment components such as dose, time and dose-time interaction. In the first case, the choice of tool dominated the windowing effect that was also very effective in detecting the miRNAs (**Figure 4A**). Once we incorporated the treatment elements, their shares decreased, but their impact was still more than treatment factors (**Figure 4B**).

### Differentially Expressed miRNAs by sRNAbench and miRDeep2

#### Concordance for sRNAbench and miRDeep2

**FIGURE 5:**
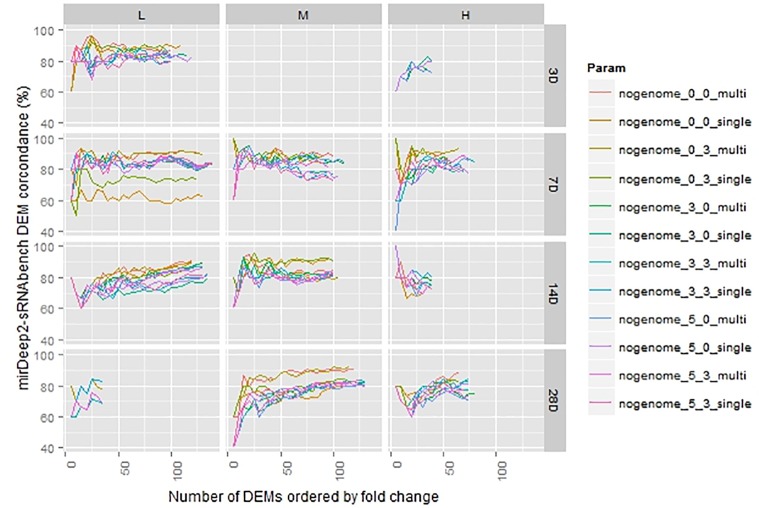
Differentially expressed miRNA comparison in sorted lists from sRNAbench and miRDeep2 under the same normalization (upper quartile).

The number of DEMs was much smaller than the detected miRNAs and, for some cases, such as 3 days with medium dose, the number dropped down to 1. In order to get reasonable statistics, we excluded those cases having less than 35 DEMs and considered only *p*-values less than 0.05 as a DEM criterion as indicated in Section “Materials and Methods.” Thus, we represented 11 treatments out of 12 in **Figure 5** in which the level of agreement between tools under the same parameters was illustrated in terms of percentages. Due to the difference in prioritization in the DEM lists acquired through miRDeep2 and sRNAbench pipelines, the overlapping ratio started at 40% in top 5 DEMs (2 DEMs were in common in top 5). As we went through the rest of the lists, we observed an increasing agreement which even reached 100% and converged around 80%.

**FIGURE 6:**
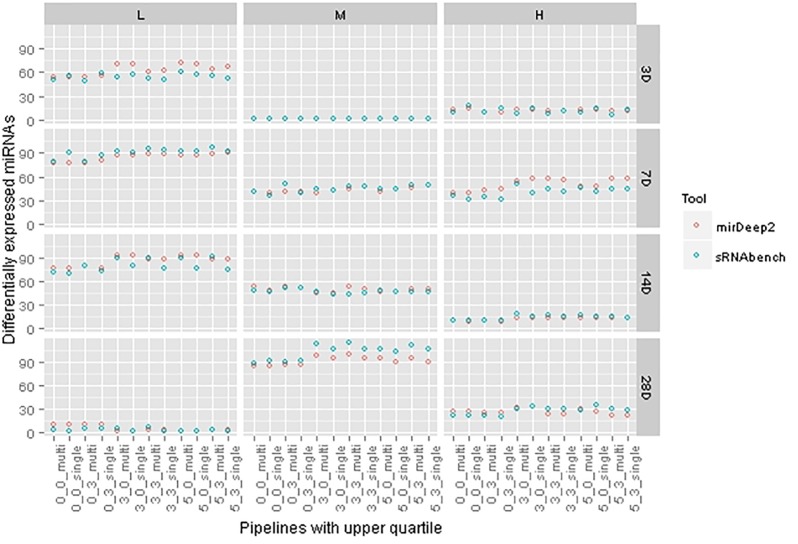
Differentially expressed miRNA set sizes for each tool under the same pipelines with upper quartile normalization.

As both tools prioritize different miRNAs either in the detection step or at the DEM computation stage, their pipelines produce nearly the same amount of DEMs whereas there was a bigger gap in their detection levels. More interestingly, we also observed treatments where sRNAbench suggests fewer DEMs than miRDeep2, although sRNAbench consistently detected more miRNAs. In **Figure 6**, we present DEM set sizes for upper quartile normalization in which DEM set sizes are close, but they do not necessarily overlap 100%. We have similar observations for other normalization choices (**Supplementary Figure S2**).

In **Figure 3A**, both tools show an increasing number of detected miRNAs as choices involve wider windows which tolerate more mismatches beyond the mature sequence. This trend is not preserved for the number of DEMs as illustrated in **Figure 6**. Indeed, there are cases, such as 14 days with medium dose, where DEM set size is almost constant even though profiling parameters change. This demonstrates the fact that miRNAs detected incrementally due to parameter change may not be differentially expressed.

#### Variance Analysis

**FIGURE 7:**
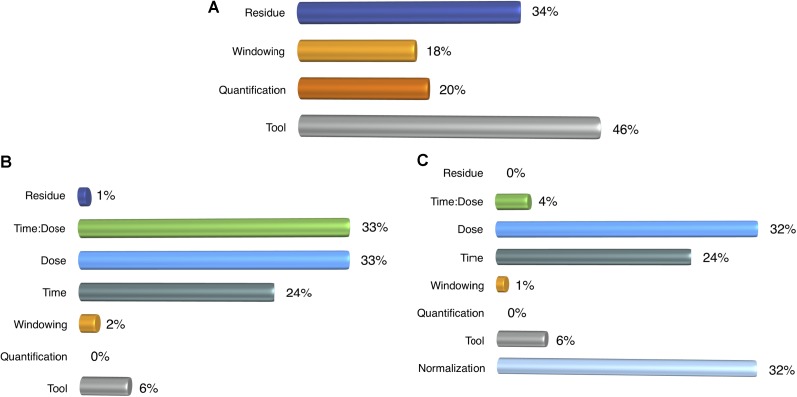
ANOVA results for the number of DEMs. **(A)** Impact of profiling parameters, **(B)** Profiling parameters and treatment components were compared and **(C)** Normalization methods were added on the top of earlier ANOVA designs.

We conducted ANOVA on the number of DEMs to have a better understanding of the parameter effect on downstream analysis. Similar to the ANOVA in the detection step, we incrementally carried out our analysis by incorporating additional parameters. In the first analysis, we only measured the impact of profiling parameters on the number of DEMs. It resulted that selection of the tool and the windowing option sustained their effects as they had in the detection rate, but ANOVA resulted in high residue (**Figure 7A**), which might be due to some other factors such as dose and time. Therefore, in the next step, we wanted to determine the effect of profiling parameters and treatment factors under a single normalization method, which was randomly chosen to be upper quartile. In **Figure 7B**, it is clearly observed that the share of the profiling parameters in the variance does not exceed 8% which indicates that the treatment has a much stronger impact on the number of DEMs and implies freedom of choice in the selection of pipeline. However, normalization introduces a huge variance that competes with that of treatment effect (**Figure 7C**). Even though profiling elements still have a minor effect on the number of DEMs, normalization drastically changes the number of DEMs as much as the dose level. Furthermore, it dominates the impact of treatment duration and therefore implies that it is a crucial step for downstream studies. When we excluded un-normalized data from our analysis, we still observed the same outcome (**Supplementary Figure S3**).

### Pairwise Concordance of Possible Bioinformatics Choices

After comparing each tool under the same parameter combinations, we also made a comparison that took into account all possible choices including choice of the tool itself as well as the normalizations for the DEM step. More specifically, we performed three types of analyses that rely on Jaccard similarities for detection rate, DEM calculation under upper quartile normalization, and DEM calculation with additional normalization choices in EdgeR.

Initially, we had 24 choices that included the selection of any tool with windowing and quantification options. For each treatment, we performed hierarchical clustering which illustrates agreement across different pipelines in terms of detection concordance. It was found that windowing was the major factor in determining the clusters of bioinformatics choices (**Supplementary Figure S4**). While bioinformatics choices having a tolerance value of 5 in the downstream were consistently clustered together regardless of their upstream end, those with exact match (0, 0) formed another cluster. Depending on the dose, bioinformatics choices with intermediate tolerance values joined one of the clusters determined by those extreme parameters. In all cases, neither sRNAbench nor miRDeep2 were split under the same parameters, showing the power of windowing parameter over all other parameters from the concordance point of view.

For the same set of parameters, we obtained clusters of bioinformatics choices based on DEM concordance for each treatment. Under the same normalization method, i.e., UQ (upper quantile), we observed that clusters were mainly built by the choice of tool and windowing (**Supplementary Figure S5**). Including 3 days with medium dose, in which we did not have a high enough number of DEMs for such analysis, pipelines belonging to the same tool form the bigger clusters and windowing determines the subclusters.

In the third step, we increased the number of bioinformatics choices by incorporating normalization methods that resulted in four times larger dendrograms than the earlier analyses. In specific, we either worked with un-normalized data (NO) or used one of the normalization methods that come with EdgeR, i.e., UQ, LRE, and TMM. The selection of a tool was still found to be a primary reason to form clusters, which implies that DEMs obtained from a tool are mostly shared by bioinformatics choices (**Supplementary Figure S6**). Even though normalization methods determine the second level of clustering for most of the cases, windowing still preserves its potential to affect the outcome of DEM calculations. Therefore, there are also clusters in which pipelines using different normalization methods are grouped together.

### Parameter Sensitivity for sRNAbench and miRDeep2

**FIGURE 8:**
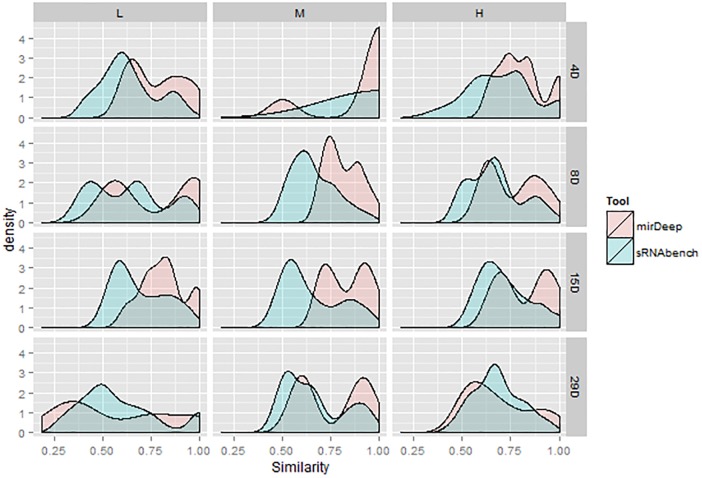
Density curves for similarity values obtained from the pipelines within each tool.

Through hierarchical clustering, we presented the Jaccard similarities of all possible parameter combinations including the tools. We further utilized these similarities for the DEM sets under upper quartile normalization to illustrate the sensitivity levels of tools for given parameter changes. We then plotted the density curves for the similarity values, which effectively outline the accumulation points. A peak around a high similarity value means that there are many parameter combinations giving very similar outcomes under the same tool. As illustrated in **Figure 8**, similarities for miRDeep2 mostly start at higher values. In addition, peaks are shifted toward the right, which might be an indication that miRDeep2 is less sensitive to parameter changes. However, when it comes to the 28th day with high dose, which is the strongest treatment, we observed similar behavior within both tools.

### Consistency in the Time-Dose Response

Both the concordance and the ANOVA results showed that downstream analysis was significantly affected by the choice of normalization. Nevertheless, the pattern in the number of DEMs across treatments needed to be investigated to elucidate the treatment effect (which we found to be competing with the normalization effect). We already discovered that number of DEMs fluctuated due to the normalization choice, but we wanted to determine whether this fluctuation would follow a pattern as we proceeded in time and dose. In order to capture a possible pattern, we computed the correlation in the number of DEMs across different time and dose combinations. In other words, we picked every possible pair of parameter combinations and compared the number of DEMs resulting from these pipelines for 12 treatment points. We found that correlation scores start from 0.92 even for the bioinformatics choices that not only differ in profiling elements, but also in their normalization step. These high correlations imply that this trend is sustained due to the treatment effect, although the numbers are drastically affected by the change in the normalization method.

## Discussion

Techniques such as qPCR and microarray have been widely used to assess the role of miRNAs in toxicogenomics research. With the advance of sequencing technologies, NGS has become a popular means for miRNA profiling and has led to novel discoveries. Thus, miRNA-seq also stands as a promising tool for toxicogenomics experiments where linking toxicants to mRNAs through miRNAs helps us understand genome-wide alterations. While miRNAs target a considerable portion of mRNAs, it is important to investigate miRNA profiling tools and their parameters to obtain a robust number of DEMs, which will potentially affect protein translation.

In a toxicological context, we examined different tools which can be run as stand-alone applications so that we could study a data set which provided a two-dimensional resolution in time and dose. Having the advantage of 12 treatment points in this particular data set, we pursued a study in which treatment effect can be a measure to analyze the miRNA-seq profiling tools. Initially, we started with four tools (miRNAkey, miRExpress, sRNAbench, and miRDeep2) whose stability was observed in terms of the number of DEMs by manipulating their own parameters. We found that miRExpress showed the most fluctuating number of DEMs as we changed its windowing (tolerance) parameter. sRNAbench and miRNAkey showed close variances, but the treatment effect was shadowed in terms of variance in the choice of miRNAkey. Thus, sRNAbench and miRDeep2 were selected to inspect the parameter effect on downstream analysis.

In our experimental setting, the number of DEMs was considered to be the indicator of the downstream effect of a toxicant. Therefore, our measurements or observations mostly relied on the change across time and dose, which was assumed to be an indicator of the treatment signal or toxic effect of thioacetamide. We then interpreted such changes to assess the magnitude of the pipeline effect on downstream analysis.

Since the detection rate is the only measure that does not need the DEMs, our first analysis relied on the number of miRNAs which had non-zero expression values. Those miRNAs, regardless of their expression levels, were considered as detected. Since the expression level was not our focus, miRNA sets from different bioinformatics choices showed high overlaps. Furthermore, ANOVA did not attribute much variance to the treatment effect. In fact, the choice of the tool played the most significant role. This observation is expected as the presence of a miRNA is not necessarily due to the toxic effect. Rather, differences in the expression levels between groups can statistically account for such an effect. For this reason, we delved deeper into DEM analysis.

In the subsequent DEM analyses, we observed that highly overlapping bioinformatics choices in the detection stage started to show differences which in turn led to decreasing agreement between bioinformatics choices. These discrepancies might be due to the selection of a profiling tool or its interaction with the parameters. Therefore, further research is warranted to investigate which tool can lead to more accurate DEMs for downstream analysis by examining the tool-specific DEMs. Current measurements, which were illustrated with the density curves in **Figure 8**, favored miRDeep2 by indicating that it is less sensitive to parameter changes. Namely, different parameter combinations resulted in very similar DEM sets for mirDeep2. Nevertheless, the same findings also emphasized that tool selection may not be that significant in the presence of a strong treatment signal. For instance, at the high dose on the 28th day, all the pipelines within each tool produce highly overlapping DEMs.

When we inspected the windowing option, we noticed an increasing number of detected miRNAs as both tools allow more mismatches. However, the number of DEMs remained almost constant and did not show considerable change beyond 3 nts. This may indicate that tolerated mismatches helped in quantifying more miRNAs with low expression values, which were not differentially expressed most of the time.

We performed ANOVA on the number of DEMs without the treatment factors, which indicated that change in DEM was not only due to the selection of the tool. Once the time and dose factors are included in the variance analysis, the treatment effect explained not only the remaining variance, but also demonstrated that the profiling elements were not that effective in downstream analysis. This finding suggests more flexibility in bioinformatics choice under the same normalization method. However, the choice of normalization can be as dominant as the treatment effect as it was shown by another ANOVA. Thus, the normalization step may require more attention than the earlier steps in miRNA-seq profiling.

The similarity between parameter combinations decreased gradually as we proceeded toward downstream analysis, especially for those that employ different normalization choices. Whereas normalization causes fluctuations in the numbers, the treatment effect enforces a pattern on these fluctuations. Namely, high correlations between parameter combinations prove the persistence of the toxic effect of thioacetamide in terms of a fluctuation pattern. Finally, the DEM sets present a diverging characteristic with lowering overlaps, but this characteristic does not attenuate the treatment signal, which is the most valuable information to be observed in an experiment.

Even though we were able to reach ∼80% overlap between DEM sets from different pipelines, one of the limitations of our study remains the lacking ground truth for toxic implications of discovered DEMs. Under this constraint, we developed our study design to evaluate different miRNA profiling tools by solely relying on the number of DEMs, which are an essential part of the downstream analysis. While different levels of discrepancies were observed between tools and protocols, focusing on the size of DEM sets and their concordances provided the opportunity to assess consistency. Namely, we not only measured the robustness of each tool, but also monitored whether the dose- and time-dependent pattern of DEM sizes would remain the same regardless of the protocol. In the absence of a ground truth set, we believe our methodology and findings provide an insight in pipeline selections not only for toxicogenomics studies, but also for other research endeavors in the community.

## Conclusion

miRNAs are important regulatory elements which are also sensitive to toxicants and require well-established bioinformatics choices for expression analysis. With a toxicogenomics focus, we studied different tools with their parameter combinations and their impact on the downstream analysis in terms of DEM sets. Our results indicated that the selection of tools and parameter manipulations could cause a limited difference in DEM sets and their variability, whereas the choice of normalization method might have more impact on concordances. We further demonstrated that the treatment effect was highly preserved despite the discordances in DEM sets, concluding that biological variance overcomes other factors in parameter choices, especially in the presence of a strong treatment signal. With different detection sensitivities, sRNAbench and miRDeep2 produced a close number of DEMs. However, mirDeep2 demonstrated lower sensitivity to parameter changes, which makes miRDeep2 preferable over other choices when windowing allows up to three nucleotides from both ends.

## Author Contributions

HB conducted all the analysis and wrote the first draft. BG contributed to the part of analysis. YW provided the dataset for this study. WT conceived the study design and finalized the manuscript. All authors read and approved the final manuscript.

## Conflict of Interest Statement

The authors declare that the research was conducted in the absence of any commercial or financial relationships that could be construed as a potential conflict of interest.

## Footnotes

1 https://www.ncbi.nlm.nih.gov/geo/query/acc.cgi?token=gpevyqegphgxbwb&acc=GSE87446

## Supplementary Material

FIGURE S1 Basic statistics for number of DEMs for each tool.

FIGURE S2 Number of detected miRNAs and DEMs for each normalization method from sRNAbench and mirDeep2 pipelines.

FIGURE S3 ANOVA on number of DEMs without normalization.

FIGURE S4 Hierarchical clusterings based on detected miRNAs from sRNAbench and mirDeep2 pipelines for 3 and 7 days.

FIGURE S5 Hierarchical clusterings based on DEMs from sRNAbench and mirDeep2 pipelines using upper quartile normalization for 3 and 7 days.

FIGURE S6 Hierarchical clusterings based on the DEMs from sRNAbench and mirDeep2 pipelines using different normalization methods.

TABLE S1 List of all pipelines with the number of detected and differentially expressed miRNAs.
