# Supplementary figures and images for: Evaluation of Bioinformatics Approaches for Next-Generation Sequencing Analysis of microRNAs with a Toxicogenomics Study Design
Source: Front Genet. 2018 Feb 6;9:22. doi: 10.3389/fgene.2018.00022 (PMC5808213; doi:10.3389/fgene.2018.00022)

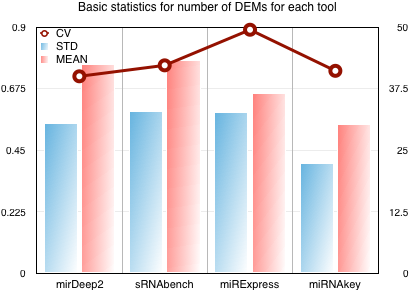

Supplement: FIGURE S1 — Basic statistics for number of DEMs for each tool. [file Image_1.tiff]

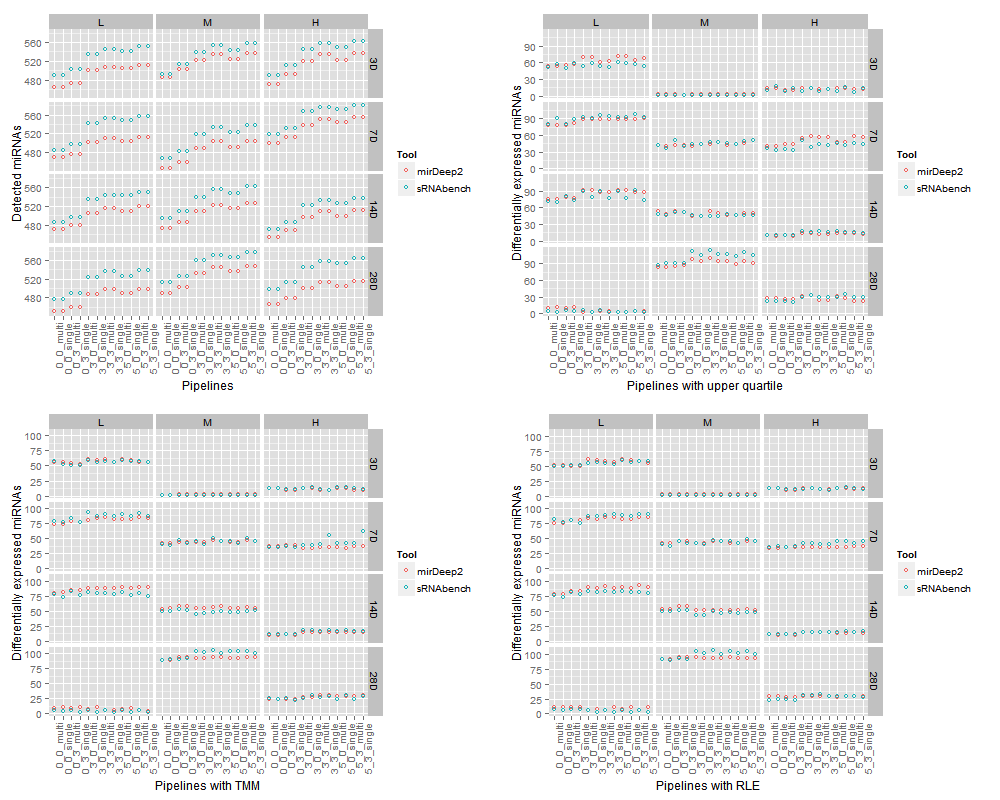

Supplement: FIGURE S2 — Number of detected miRNAs and DEMs for each normalization method from sRNAbench and mirDeep2 pipelines. [file Image_2.tiff]

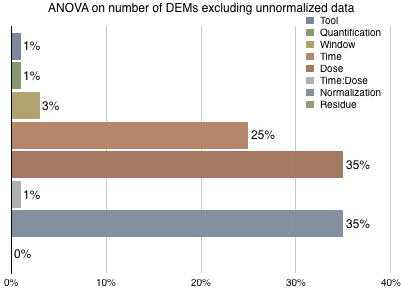

Supplement: FIGURE S3 — ANOVA on number of DEMs without normalization. [file Image_3.tiff]

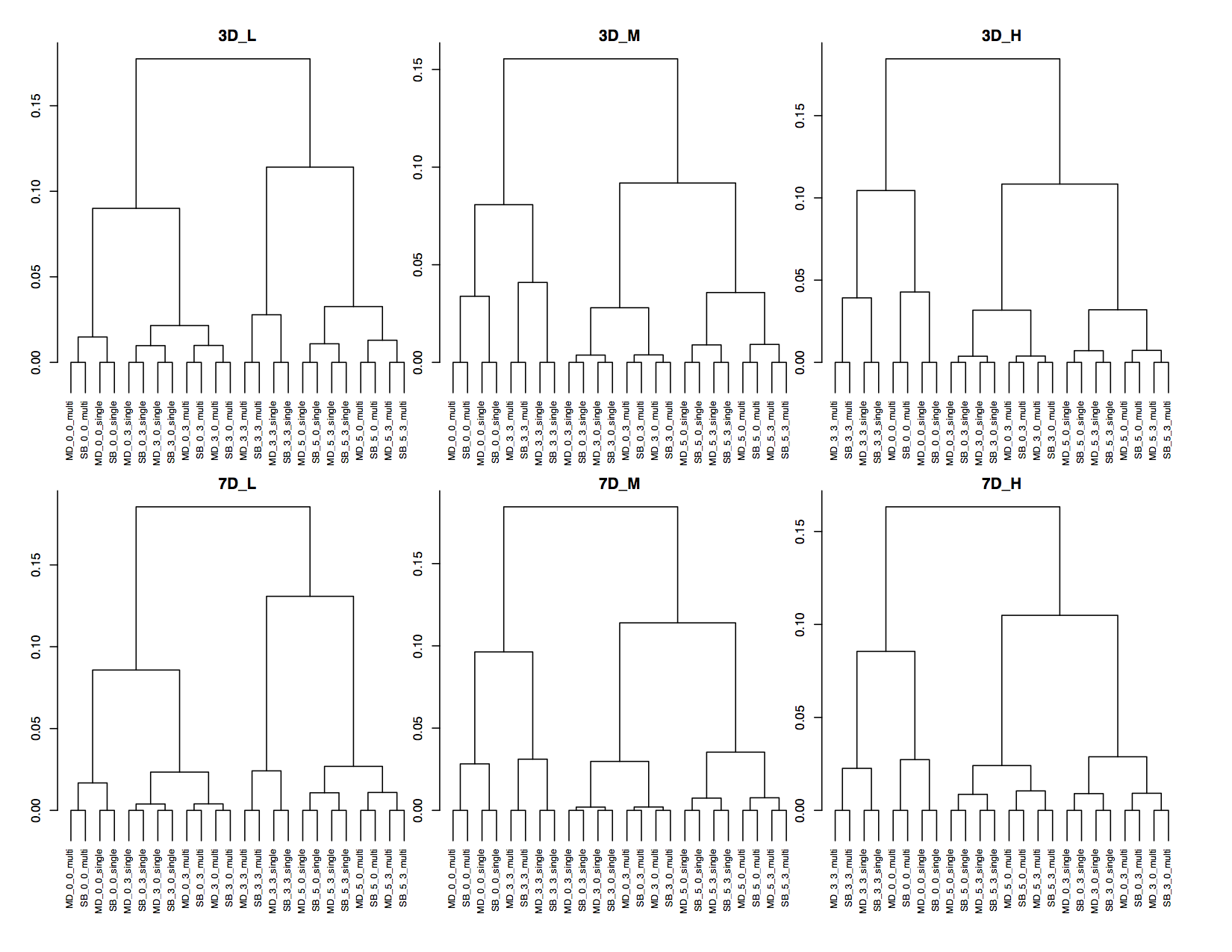

Supplement: FIGURE S4 — Hierarchical clusterings based on detected miRNAs from sRNAbench and mirDeep2 pipelines for 3 and 7 days. [file Image_4.tiff]

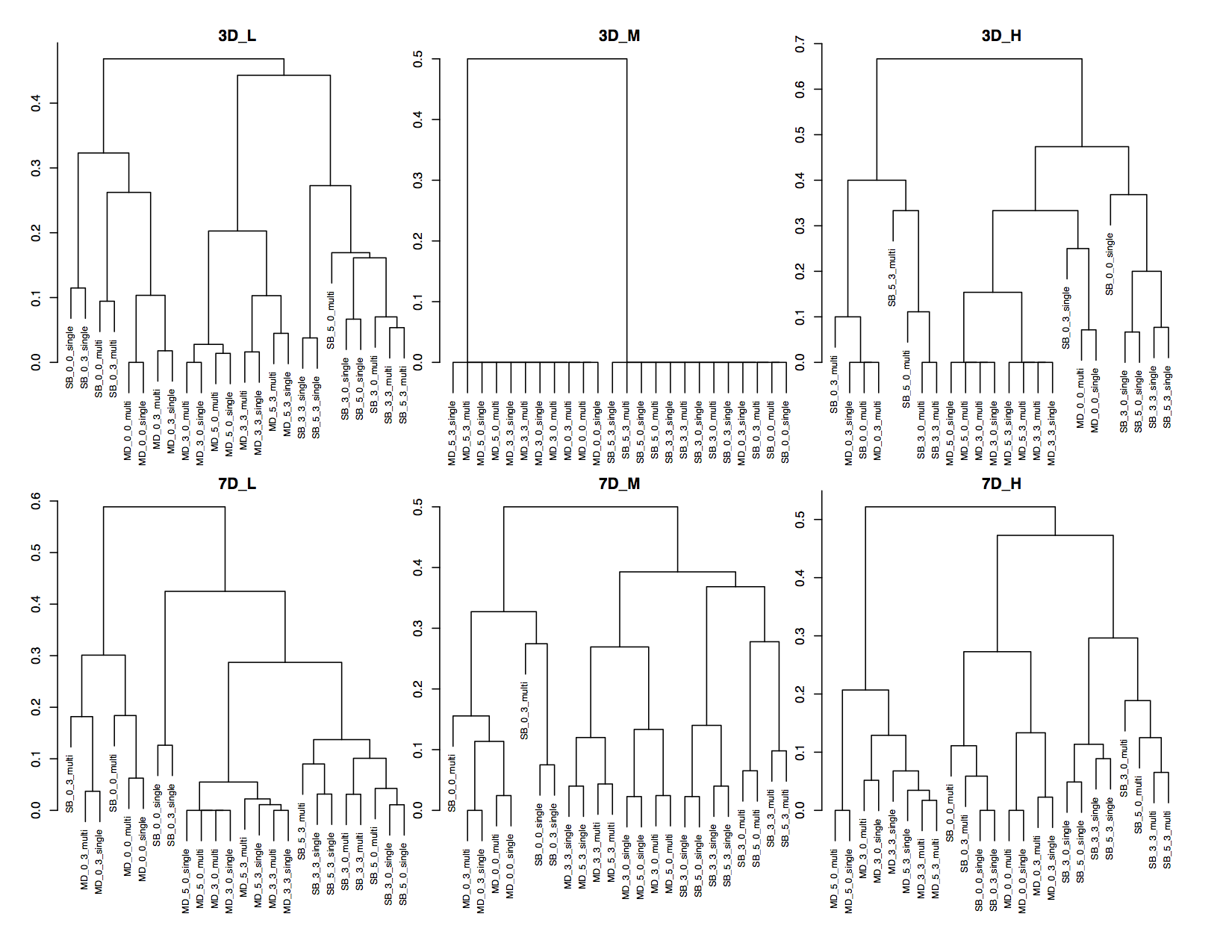

Supplement: FIGURE S5 — Hierarchical clusterings based on DEMs from sRNAbench and mirDeep2 pipelines using upper quartile normalization for 3 and 7 days. [file Image_5.tiff]

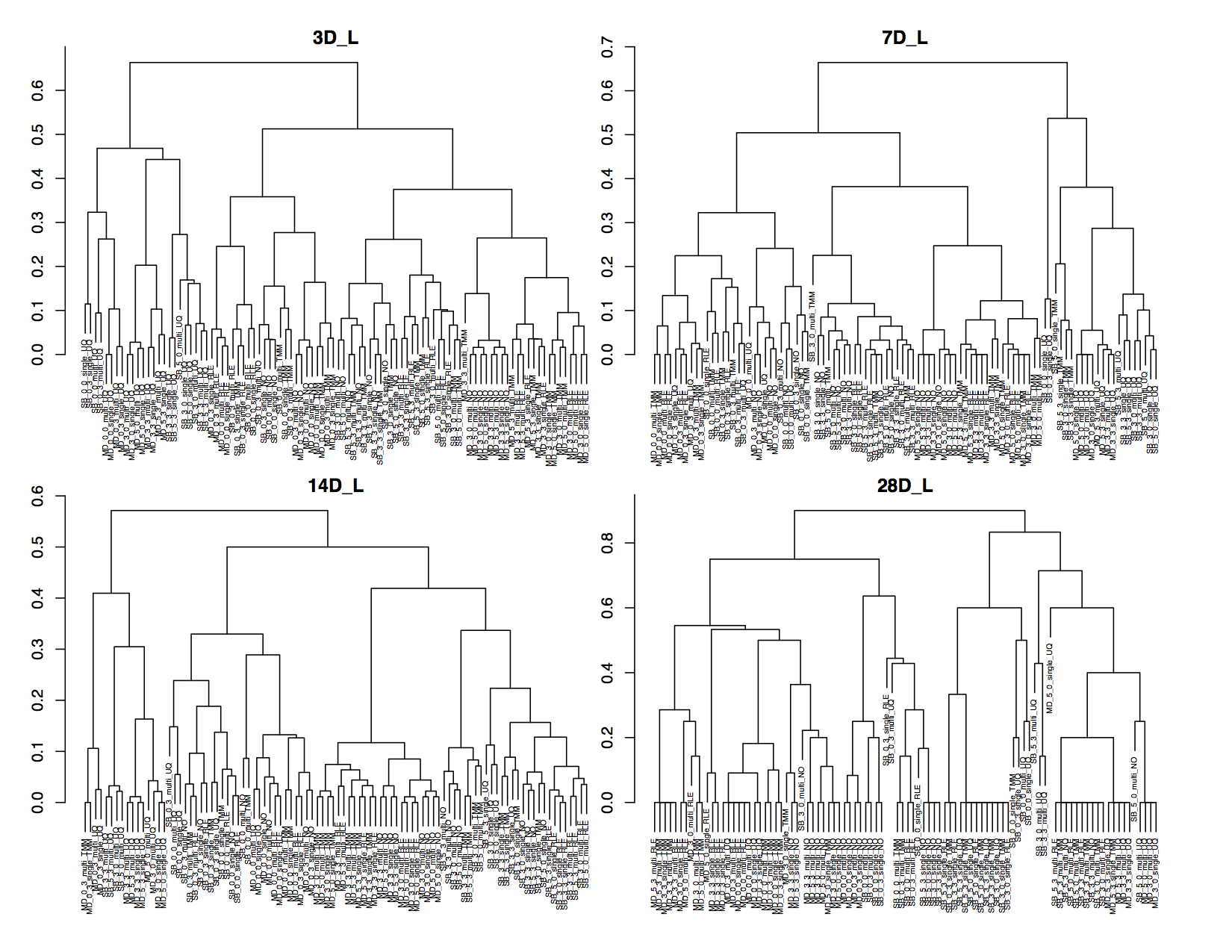

Supplement: FIGURE S6 — Hierarchical clusterings based on the DEMs from sRNAbench and mirDeep2 pipelines using different normalization methods. [file Image_6.tiff]
